# Serine-arginine protein kinase 1 (SRPK1) promotes EGFR-TKI resistance by enhancing GSK3β Ser9 autophosphorylation independent of its kinase activity in non-small-cell lung cancer

**DOI:** 10.1038/s41388-023-02645-2

**Published:** 2023-03-03

**Authors:** Jing-Qiang Huang, Ling-Xin Duan, Qiu-Yu Liu, He-Feng Li, Ao-Ping Hu, Jun-Wei Song, Chuxuan Lin, Bingsheng Huang, Da Yao, Bin Peng, Yehong Sun, Yuxin Wen, Lin Yang, Xingzhi Xu, Li-Yun Gong

**Affiliations:** 1 Guangdong Provincial Key Laboratory for Genome Stability and Disease Prevention, Department of Biochemistry and Molecular Biology, School of Basic Medical Sciences, Shenzhen University Medical School, Shenzhen, Guangdong 518055 P.R. China; 2 Guangdong Provincial Key Laboratory for Genome Stability and Disease Prevention, Carson International Cancer Center, and Marshall Laboratory of Biomedical Engineering, Shenzhen University Medical School, Shenzhen, Guangdong 518055 P.R. China; 3 School of Biomedical Engineering, Shenzhen University Medical School, Shenzhen, Guangdong 518055 P.R. China; 4 School of Pharmaceutical Sciences, Shenzhen University Medical School, Shenzhen, Guangdong 518055 P.R. China; 5 Department of Pathology, Henan Provincial People’s Hospital, The People’s Hospital of Zhengzhou University, Zhengzhou, Henan 450003 P.R. China; 6 Medical School, Southern University of Science and Technology, Shenzhen, Guangdong 518005 P.R. China; 7 Medical AI Lab, School of Biomedical Engineering, Shenzhen University Medical School, 518055 Shenzhen, Guangdong P.R. China; 8 Department of Thoracic Surgery, The First Affiliated Hospital of Shenzhen University, Shenzhen Second People’s Hospital, Shenzhen, P.R. China; 9 Department of Clinical Pharmacy, Shenzhen Traditional Chinese Medicine Hospital, 4th Clinical Medical College of Guangzhou University of Chinese Medicine, Shenzhen, Guangdong 518005 P.R. China; 10 Department of Thoracic Surgery, Shenzhen People’s Hospital, 2nd Clinical Medical College of Jinan University, Shenzhen, Guangdong 518020 P.R. China

**Keywords:** Non-small-cell lung cancer, Cancer therapeutic resistance

## Abstract

Resistance to epidermal growth factor receptor (EGFR) tyrosine kinase inhibitors (TKIs) is a major challenge for clinicians and patients with non-small cell lung cancer (NSCLC). Serine-arginine protein kinase 1 (SRPK1) is a key oncoprotein in the EGFR/AKT pathway that participates in tumorigenesis. We found that high SRPK1 expression was significantly associated with poor progression-free survival (PFS) in patients with advanced NSCLC undergoing gefitinib treatment. Both in vitro and in vivo assays suggested that SRPK1 reduced the ability of gefitinib to induce apoptosis in sensitive NSCLC cells independently of its kinase activity. Moreover, SRPK1 facilitated binding between LEF1, β-catenin and the EGFR promoter region to increase EGFR expression and promote the accumulation and phosphorylation of membrane EGFR. Furthermore, we verified that the SRPK1 spacer domain bound to GSK3β and enhanced its autophosphorylation at Ser9 to activate the Wnt pathway, thereby promoting the expression of Wnt target genes such as Bcl-X. The correlation between SRPK1 and EGFR expression was confirmed in patients. In brief, our research suggested that the SRPK1/GSK3β axis promotes gefitinib resistance by activating the Wnt pathway and may serve as a potential therapeutic target for overcoming gefitinib resistance in NSCLC.

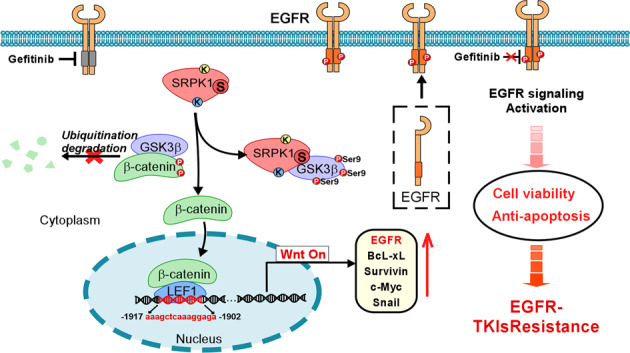

## Introduction

Non-small-cell lung cancer (NSCLC), which accounts for 80% of all diagnosed lung cancer cases, was the leading cause of cancer-associated mortality worldwide in 2021 [1, 2]. Aberrant activation of the epidermal growth factor receptor (EGFR/ERBB1) contributes to lung cancer [3, 4]. Increased levels of EGFR expressed on cellular membranes leads to transphosphorylation, receptor activation, and EGFR signal emission [5]. Ligand-dependent EGFR activation or ligand-independent constitutive EGFR activation triggers several downstream signaling pathways that inhibit apoptosis [6, 7]. EGFR tyrosine kinase inhibitors (TKI) such as gefitinib have been widely used to treat patients with EGFR-activating mutations [8–10]. Unfortunately, the long-term efficacy of TKI is limited by the acquisition of TKI resistance in 80–90% of these patients within one year [11, 12]. The mechanisms underlying the acquisition of resistance to EGFR-TKI include genetic mutation of the EGFR, activation of bypass signaling, and histological/phenotypic transformations [13–16], although the intrinsic mechanisms underlying EGFR-TKI resistance remain unclear. Therefore, it is necessary to elucidate the molecular basis of EGFR overexpression and hyperactivation, which will contribute to the development of more effective strategies to overcome EGFR-TKI resistance and improve clinical outcomes.

Serine-arginine protein kinase 1 (SRPK1) is a member of the SR kinase family that contains a spacer region dividing its kinase domain into two parts [17] comprising the kinase domains I–VI and VII–XI. SRPK1 and its downstream targets are associated with numerous pathological and biological processes [18, 19]. For example, inhibition of the kinase function of SRPK1 leads to a marked switch from short to long bromodomain-containing 4 (BRD4) isoforms, resulting in cell cycle arrest [20]. SRPK1 also initiates parental genome reprogramming by catalyzing the phosphorylation of protamine in fertilized eggs [21]. Furthermore, during tumor angiogenesis, SRPK1 maintains the balance between the expression of the pro-angiogenic vascular endothelial growth factor (VEGF)-A165 and the anti-angiogenic VEGF-A165b [22]. However, previous studies have primarily focused on the splicing activity of SRPK1, with few studies assessing its function as a scaffold oncoprotein during tumor progression and the underlying mechanisms. Notably, SRPK1 is known to participate in EGF-induced EGFR activation and interact with protein kinase B (AKT) to regulate AKT phosphorylation [23, 24]. In addition, our group reported that aberrant SRPK1 expression promotes stem cell-like phenotypes in NSCLC cells by activating the Wnt/β-catenin pathway [25]. EGFR is a direct target gene of Wnt signaling [26], and aberrant Wnt signaling is known to be involved in neoplasia and drug resistance in multiple types of cancer [27]. EGFR-dependent cancers have a unique dependence on Wnt signaling following EGFR inhibition [28], suggesting that SRPK1 is a key node linking EGFR with the Wnt pathway. However, the role of SRPK1 in EGFR-TKI resistance remains unclear and warrants further investigation.

In the present study, we found that high SRPK1 expression was associated with poor progression-free survival (PFS) and EGFR expression in patients with advanced NSCLC. SRPK1 can promote glycogen synthase kinase-3 (GSK3β) autophosphorylation at Ser9 in a manner that is independent of its kinase activity, which ultimately confers gefitinib resistance in NSCLC by activating Wnt/β-catenin signaling. These results may provide a theoretical and experimental basis for the further development of EGFR-TKI therapy for NSCLC.

## Results

### SRPK1 overexpression is associated with gefitinib resistance in NSCLC patients

**Fig. 1:**
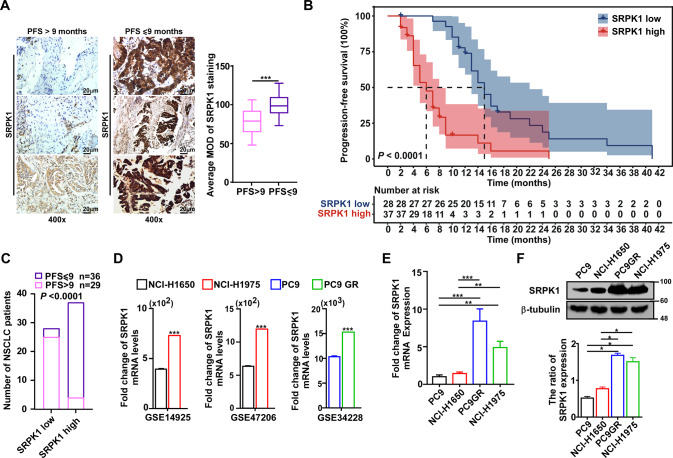
SRPK1 overexpression is associated with gefitinib resistance in NSCLC patients. **A** Representative images of IHC staining of SRPK1 in lung tissue sections from NSCLC patients (left; *n* = 65), and quantitative analysis of SRPK1 staining (right); scale bars: 20 μm. Data represent the mean optical density (MOD) ± SD (*n* = 10, 2-tailed Student’s *t* test). The whiskers represent 5 and 95 percentiles. **B** Kaplan-Meier curves for estimation of PFS in NSCLC patients with high- or low- SRPK1 expression (Log-rank test). **C** Pearson chi-squared (χ^2^) test analysis of the relationship between PFS and SRPK1 expression; the number of NSCLC patients is shown. **D** Analyses of *SRPK1* gene expression in NSCLC cell lines in publicly available datasets (NCBI/GEO/GSE 14925, NCBI/GEO/GSE 47206, and NCBI/ GEO/GSE 34228). **E** qRT-PCR analysis and (**F**) Western blot analysis of SRPK1 expression in PC9, NCI-H1650, PC9GR, and NCI-H1975 cells. Data represent the mean ± SD (*n* = 3). ^*^P < 0.05, ^**^P < 0.01^, ***^P < 0.001.

We analyzed SRPK1 expression in tissues obtained from 65 NSCLC patients with EGFR mutations and classified the staining scores into two groups according to the optimal cut-off value from ROC curve analysis. After grouping patient tissue sections by median progression-free survival (PFS), we found that SRPK1 expression was significantly increased in the PFS ≤ 9 group compared with that in the PFS > 9 group (Fig. 1A and Table S1). Kaplan-Meier curves and log-rank tests showed a significant difference in PFS between the high- and low- SRPK1 expression groups (*P* < 0.001; Fig. 1B). Moreover, Pearson’s χ^2^ tests revealed that high SRPK1 expression was closely associated with reduced PFS (Fig. 1C). Thus, our results suggested that high SRPK1 expression is correlated with poor PFS in NSCLC patients undergoing EGFR-TKI treatment. Furthermore, we found that SRPK1 expression levels were significantly upregulated by 1.8-fold in NCI-H1975 compared with NCI-H1650 and 1.5-fold in PC9GR compared with PC9 in publicly available datasets (NCBI/GEO/GSE 14925, NCBI/GEO/GSE 47206, and NCBI/ GEO/GSE 34228) and confirmed at the both mRNA and protein levels (Fig. 1 D-F). Taken together, these results indicated that high SRPK1 expression correlates with poor PFS and gefitinib resistance in NSCLC patients with EGFR mutations.

### Upregulation of SRPK1 inhibits gefitinib-induced apoptosis in NSCLC cell lines

**Fig. 2:**
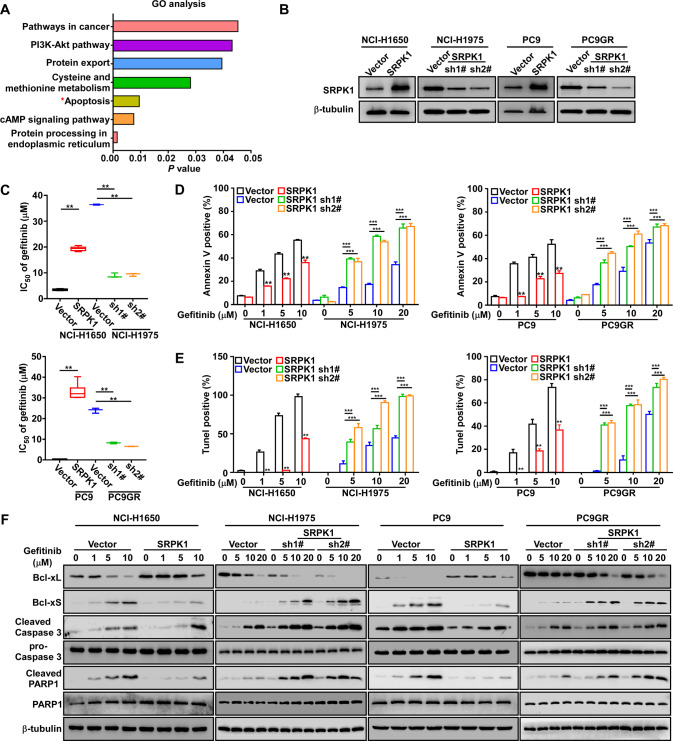
SRPK1 upregulation inhibits gefitinib-induced apoptosis in NSCLC cell lines. **A** GO analysis of proteins enriched by SRPK1 antibodies after IP-MASS assays revealed the biological processes associated with SRPK1 interactions. **B** Western blot analysis confirmed SRPK1 overexpression in gefitinib-sensitive NCI-H1650 and PC9 cells, and SRPK1 downregulation in gefitinib-resistant NCI-H1975 and PC9GR cells. **C** Verification of IC_50_ values at a range of gefitinib concentrations (0, 0.01, 0.1, 2,5, 10, 20, 30, 40 µM) in the indicated cells (*n* = 3). The percentages of (**D**) apoptotic and (**E**) TUNEL^+^ cells following gefitinib treatment. **F** Bcl-xL, Bcl-xS, cleaved caspase-3, and cleaved PARP1 protein expression in the indicated cell lines following treatment with gefitinib (0, 1, 5, 10, 20 µM); β-tubulin serves as a loading control. Data represent the mean ± SD (*n* = 3). ^**^P < 0.01, ^***^P < 0.001.

Gene ontology (GO) analysis of the HA-SRPK1 interactome revealed that apoptosis-related proteins were enriched (Fig. 2A and Table S6). We stably upregulated SRPK1 expression in gefitinib-sensitive NCI-H1650 and PC9 cells and downregulated SRPK1 expression in gefitinib-resistant NCI-H1975 and PC9GR cells (Fig. 2B and S2A). The IC_50_ value of gefitinib increased in SRPK1-overexpressed cells and decreased in SRPK1 knockdown cells (Fig. 2C). These results suggested that SRPK1 increased gefitinib resistance in gefitinib-sensitive cells, whereas inhibiting SRPK1 expression restored the sensitivity of gefitinib-resistant cells. This occurred independently of the EGFR secondary resistance mutations.

Next, Annexin V and TUNEL assays revealed that, in PC9 and NCI-H1650 cells overexpressing SRPK1, the percentages of Annexin V^+^ and TUNEL^+^ cells were significantly reduced in the gefitinib-treated group compared with those in the control group, while the opposite effects were observed after SRPK1 downregulation (Fig. 2D, E, and S1A, B). In accordance with these findings, Western blot analysis revealed that Bcl-xL expression was increased, while expression of pro-apoptosis proteins was decreased in SRPK1-overexpressed cells; the opposite effects were observed in SRPK1 knockdown cells (Fig. 2F and S2C–E). Furthermore, the apoptosis regulator Bcl-X exists as two isoforms with opposing functions generated by alternative splicing. In SRPK1-overexpressing cells, expression of the anti-apoptotic isoform (Bcl-xL) was increased, while expression of the pro-apoptotic isoform (Bcl-xS) was decreased; the opposite effects were observed in SRPK1 knockdown cells. Our results indicated that SRPK1-mediated alternative splicing of Bcl-X is involved in promoting gefitinib resistance.

In addition, we found that gefitinib did not affect SRPK1 expression in NCI-H1650 and NCI-H1975 cells, whereas SRPK1 expression was decreased in PC9 and PC9GR cells following gefitinib treatment (Fig. S2B). However, SRPK1 overexpression inhibited the gefitinib-induced reduction in SRPK1 in PC9 cells (Fig. S2B). Even after gefitinib treatment, SRPK1 expression levels in the overexpression group were consistently higher than those in the control group, while SRPK1 expression levels in the knockdown group were maintained at lower levels (Fig. S2B). These data suggested that the anti-apoptotic effects of SRPK1 are related to its expression levels.

Collectively, these results indicated that aberrant SRPK1 expression induces gefitinib resistance by inhibiting apoptosis in NSCLC cells.

### SRPK1 ablation mediates tumor growth inhibition in vivo in EGFR mutant tumors

**Fig. 3:**
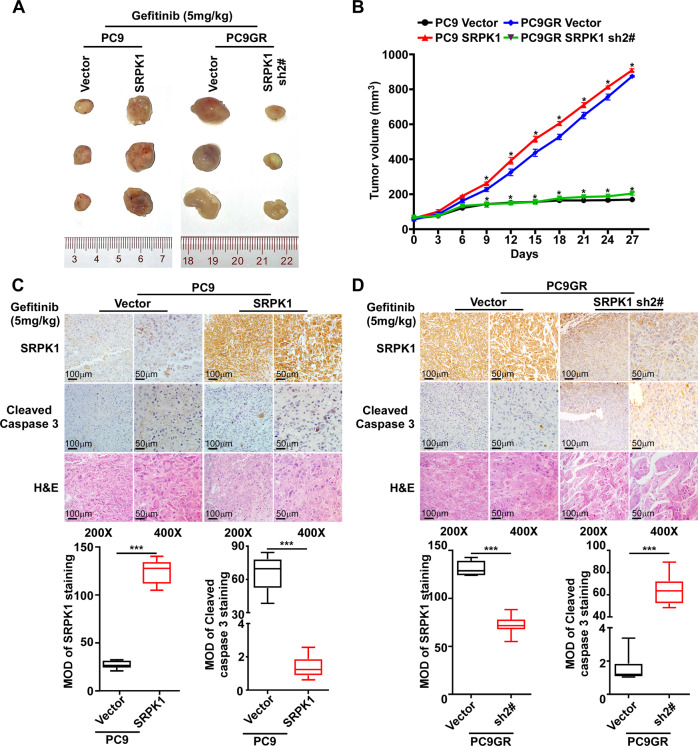
SRPK1 ablation mediates tumor growth inhibition in EGFR mutant tumors in vivo. **A** Subcutaneous tumors at the experimental endpoint. **B** Growth curves of xenograft tumors derived from PC9-vector, PC9-SRPK1, PC9GR-Vector, and PC9GR-SRPK1-sh2# cells in mice treated with gefitinib (*n* = 5). **C** IHC analysis of SRPK1 and cleaved caspase 3 levels in xenograft tumors derived from PC9-vector and PC9-SRPK1 and (**D**) PC9GR-Vector and PC9GR-SRPK1-sh2# cells in mice treated with gefitinib. Statistical analysis of staining is provided. Data represent the mean optical density (MOD) ± SD (*n* = 10). The whiskers represent 5 and 95 percentiles. ^*^P < 0.05, ^**^P < 0.01^, ***^P < 0.001.

To evaluate the effects of SRPK1 on gefitinib resistance in vivo, we subcutaneously inoculated cells mixed with Matrigel into the inguinal fold of BALB/c nude mice. After treatment with gefitinib, xenograft tumors derived from PC9-SRPK1 cells showed a marked increase in tumor growth rate and mass compared with those derived from PC9-Vector cells (Fig. 3A, B). Conversely, xenograft tumors derived from PC9GR-SRPK1-sh2# cells exhibited a marked decrease in tumor growth rate and mass compared with those in the control group (Fig. 3A, B). IHC analysis also revealed that cleaved caspase-3 levels were reduced in xenograft tumors produced from *SRPK1*-overexpressing cells, and increased in those produced from *SRPK1*-knockdown cells (Fig. 3C, D). These results suggested that the SRPK1 depletion is indispensable for apoptosis gene expression and EGFR mutant tumor growth inhibition under TKI treatment.

### SRPK1 rescues the reduction of GSK3β Ser9 phosphorylation following gefitinib treatment, independent of its kinase activity

**Fig. 4:**
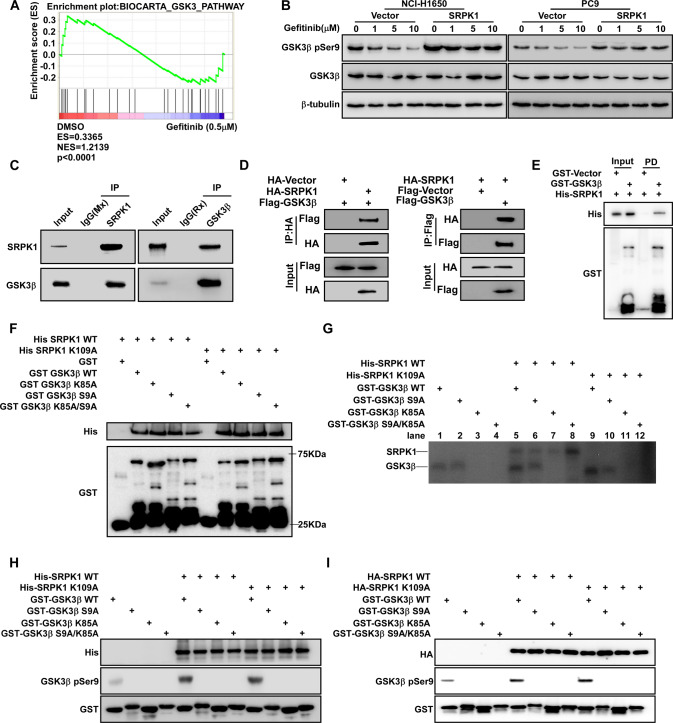
SRPK1 rescues the reduction of GSK3β Ser9 phosphorylation under gefitinib treatment, independent of its kinase activity. **A** GSEA showing a significant association between gefitinib treatment and GSK3 phosphorylation (BIOCARTA_GSK3_PATHWAY) in the GSE75309 database. **B** Western blot analysis of GSK3β pSer9 expression in cells treated with gefitinib (0, 1, 5, or 10 µM). **C** IP analysis of the interactions between SRPK1 and GSK3β examined in NCI-H1975 cells. **D** Co-IP analysis of the interaction between SRPK1 and GSK3β in 293T cells transfected with HA-Vector, HA-SRPK1, Flag-Vector or Flag-GSK3β, as indicated. **E** Pulldown assay investigation of the interaction between SRPK1 WT and GSK3β WT in vitro. **F** Pulldown assay investigation of the interaction between SRPK1 and GSK3β variants. **G** Isotope radiation assay of GSK3β Ser9 phosphorylation status in SRPK1 and GSK3β variants co-incubated in isotope radiation kinase assay buffer for 30 min at 30 °C. **H** Kinase activity assay of the in vitro phosphorylation relationship between SRPK1 and GSK3β in *E. coli* (BL21 DE3) cells transfected with His-SRPK1 WT, His-SRPK1 K109A, GST-GSK3β WT, GST-GSK3β S9A, GST-GSK3β K85A, and GST-GSK3β S9A/K85A. **I** Kinase activity assay of the in vivo phosphorylation relationship between SRPK1 and GSK3β precipitated from lysates of 293T cells expressing tagged peptides (HA-SRPK1 WT and HA-SRPK1 K109A) using HA antibody.

We performed GSEA analysis containing only PC9 cells before and after gefitinib treatment in the GSE75309 cohort. GSEA revealed that GSK3β signaling pathway activity was significantly decreased in samples following gefitinib treatment (Fig. 4A). Moreover, we found that GSK3β Ser9 phosphorylation was increased at the basal level in PC9GR and NCI-H1975 compared with that in gefitinib-sensitive cells (PC9 and NCI-H1650) (Fig. S3A). We further observed that GSK3β Ser9 phosphorylation level decreased following gefitinib treatment in a dose-dependent manner (Fig. 4B and S3B). Furthermore, SRPK1 overexpression increased GSK3β pSer9 levels, whereas SRPK1 knockdown further decreased GSK3β pSer9 levels (Fig. 4B and S3B). Co-IP assays revealed that both endogenous and epitope-tagged SRPK1 interacted with GSK3β (Fig. 4C, D). GST-pulldown assays showed that SRPK1 interacted with GSK3β directly (Fig. 4E). In addition, GST-GSK3β, a kinase-dead mutant GST-GSK3β (K85A), a phosphorylation-defective mutant GST-GSK3β (S9A), and the double mutant GST-GSK3β (S9A/K85A) pulled down His-SRPK1 and a kinase-dead mutant His-SRPK1(K109A) (Fig. 4F). This indicated that SRPK1 interacts directly with GSK3β, in a manner that is independent of its kinase activity. Next, we investigated whether GSK3β serves as a substrate for SRPK1. In vitro kinase assays revealed auto-phosphorylation (upper radioisotope signal) of SRPK1 in His-SRPK1, but not in His-SRPK1(K109A), indicating that the K109A mutation blocks the kinase activity of SRPK1 (Fig. 4G, lanes 5–8 and 9–12). Regardless of SRPK1 kinase activity, enhanced radioisotope signals (under signal) were detected in GST-GSK3β and weaker signals were detected in GST-GSK3β (S9A), while no signals were detected in the other groups (Fig. 4G). These results demonstrated that GSK3β is not a substrate for SRPK1 and this was further confirmed by in vitro kinase assays (Fig. 4H, I), indicating that SRPK1 enhances GSK3β Ser9 autophosphorylation independently of its kinase activity, which may in turn increase resistance to gefitinib in NSCLC.

To rule out the impact of AKT on GSK3β phosphorylation, we incubated PC9 and NCI-H1650 cells with the AKT inhibitor triciribine. GSK3β phosphorylation was upregulated in *SRPK1*-overexpressed cells following AKT inhibitor treatment (Fig. S3C and S3D), suggesting that SRPK1-mediated GSK3β phosphorylation occurs in an AKT-independent manner.

### The SRPK1 spacer domain is required for gefitinib resistance, while kinase activity is not

**Fig. 5:**
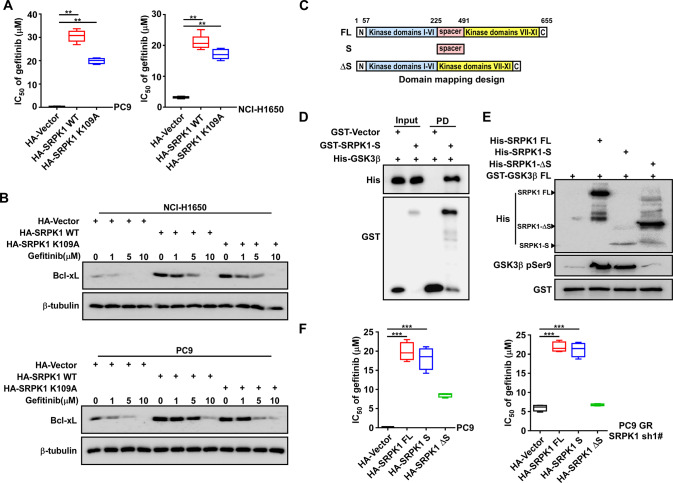
SRPK1 spacer domain is required for gefitinib resistance, while kinase activity is not. **A** IC_50_ for gefitinib in NCI-H1650 and PC9 cells overexpressing SRPK1 WT, SRPK1 K109A, or vector only. **B** Western blot analysis of Bcl-xL protein levels. **C** Schematic of the SRPK1 fragment domains. **D** Pulldown assay to investigate the interaction between GST-SRPK1-S and His-GSK3β WT in vitro (S: spacer domain). **E** Kinase activity assay of the phosphorylation relationship between SRPK1 fragments and GSK3β in vitro (S: spacer domain). **F** IC_50_ for gefitinib in PC9 and *SRPK1*-silenced PC9GR cells transfected with the indicated plasmids. Data represent the mean ± SD (*n* = 6). The whiskers represent 5 and 95 percentiles. **P < 0.01.

Next, we explored whether SRPK1 functions as a scaffold protein rather than a kinase to confer gefitinib resistance. MTT assays showed that the IC_50_ values of gefitinib in the SRPK1-WT and the SRPK1-K109A groups were similarly increased (Fig. 5A). Next, we found that the expression of Bcl-xL was increased in both the SRPK1-WT and SRPK1-K109A groups after gefitinib treatment (Fig. 5B). These results demonstrated that the function of SRPK1 in gefitinib resistance is independent of its kinase activity.

We further investigated which SPRK1 domain interacts with GSK3β to promote GSK3β Ser9 autophosphorylation and, ultimately, gefitinib resistance. We generated truncated mutants of SRPK1ΔN, SRPK1ΔS, SRPK1ΔK1, SRPK1-S and SRPK1ΔK2 (Fig. 5C and S4A). GST-pulldown assays demonstrated that SRPK1 binds to GSK3β through its spacer domain (Fig. 5D and S4A). In vitro kinase assays revealed upregulation of GSK3β Ser9 autophosphorylation following expression of SRPK1-FL or SRPK1-S and this effect was diminished following expression of SRPK1ΔS (Fig. 5E). In addition, we found that the nuclear expression of SRPK1 was increased in PC9GR cells compared to PC9 cells, suggesting that increasing SRPK1 levels promote SRPK1 translocation into the nucleus (Fig. S4B). However, while gefitinib promoted SRPK1 translocation into the nucleus in PC9 cells, SRPK1 was translocated into the cytoplasm following gefitinib treatment of PC9GR cells (Fig. S4B), indicating that the sensitivity of gefitinib may be related to cytoplasmic localization of SRPK1. We also found that the spacer domain contributes to the protein-protein interaction between SRPK1 and GSK3β, but not SRPK1 sub-cellular localization (Fig. 5D, S4A and S4C). Moreover, expression of either SRPK1-FL or SRPK1-S rescued the growth of SRPK1-deficient cells under gefitinib treatment (Fig. 5F).

To explore whether the interaction between SRPK1 and GSK3β is dependent on its kinase activity, we treated NCI-H1975 cells with SPHINX31. IP assays showed that SPHINX31 did not reduce the interaction between SRPK1 and GSK3β (Fig. S4D). Furthermore, SRPK1 inhibitors (SRPIN340 and SPHINX31) did not increase the sensitivity of NCI-H1975, PC9 SRPK1 and PC9GR cells to gefitinib (Fig. S4E), suggesting that SRPK1 kinase activity is not involved in EGFR-TKI resistance in these cells.

Taken together, these results indicated that SRPK1 spacer domain acts as a limiting factor for inhibition by gefitinib. Thus, it can be hypothesized that SRPK1-GSK3β axis promotes gefitinib resistance in NSCLC, with the catalytic function being dispensable and the spacer domain required for initiation.

### SRPK1 promotes the binding between β-catenin and the EGFR promoter region to increase mEGFR accumulation and phosphorylation

**Fig. 6:**
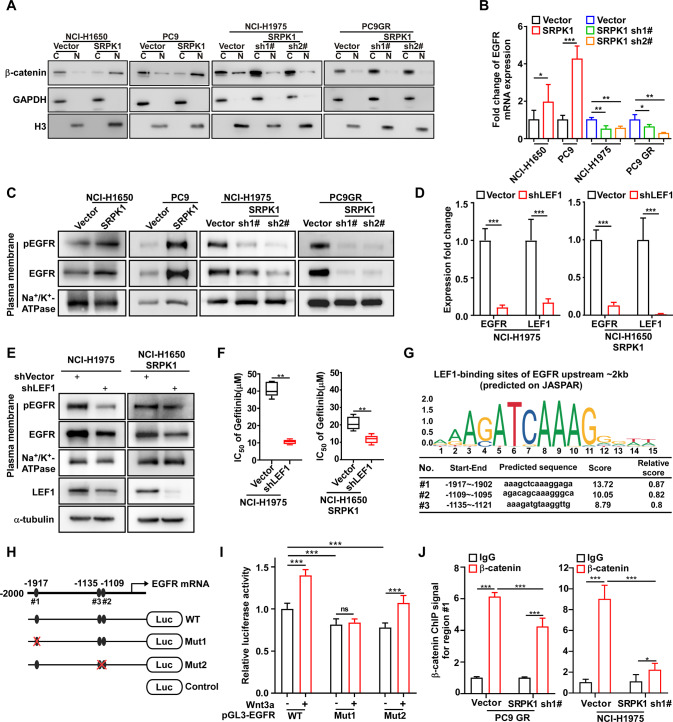
SRPK1 promotes binding of β-catenin to the EGFR promoter region to increase mEGFR accumulation and phosphorylation. **A** Western blot analysis of β-catenin expression in the nuclear fractions. **B** qRT-PCR analysis of EGFR mRNA expression; data represent the mean ± SD (*n* = 3). **C** Western blot analysis of the expression and phosphorylation of membrane EGFR; Na^+^/K^+^-ATPase was used as a loading control. **D** qRT-PCR analysis of EGFR mRNA expression. **E** Western blot analysis of mEGFR expression after LEF1 knockdown in SRPK1-transduced NCI-H1650 cells and SRPK1-silenced NCI-H1975 cells. **F** IC_50_ of gefitinib verified in LEF1-silenced cells. ^*^P < 0.05, ^**^P < 0.01, ^***^P < 0.001. **G** The LEF1 binding regions (Region#1–3) in the 2-kb upstream promoter region of *EGFR* predicted by the JASPAR website. **H** Schematic diagram of the luciferase reporter plasmids containing wild-type and mutant predicted LFF1 binding regions, and (**I**) Luciferase assay after transfection into 293T cells (with or without Wnt3a treatment). **J** ChIP-qPCR assays performed with anti-β-catenin antibody and primers across region#1 in SRPK1-silenced or vector-only PC9 GR and NCI-H1975 cells.

Next, we further investigated the molecular mechanisms by which SRPK1 induced gefitinib resistance. GO analysis revealed that canonical Wnt signaling was activated in PC9GR cell, and GSEA analysis showed that Wnt signaling was significantly associated with gefitinib treatment (Fig. S5A-B). Luciferase and real-time PCR assays confirmed that SRPK1 upregulation significantly activated the Wnt/β-catenin pathway (Fig. S5C and S5E), which were suppressed by β-catenin knockdown, suggested that these genes are downstream targets of the Wnt/β-catenin pathway (Fig. S5E). Western blot analysis showed that SRPK1 did not regulate total β-catenin protein levels in the SRPK1 overexpression and deficient cells (Fig. S6A). We observed that SRPK1 overexpression promoted the nuclear translocation of β-catenin, whereas SRPK1 depletion inhibited this process (Fig. 6A and Fig. S5D, S6B). Furthermore, we used XAV939 (β-catenin inhibitor) to determine whether SRPK1 overexpression reversed gefitinib resistance in PC9 cells. The data showed that XAV939 did not further increase the sensitivity of gefitinib in PC9 SRPK1 cells (Fig. S6C). Moreover, we found that XAV939 treatment is not able to induce β-catenin degradation in SRPK1-overexpressing cells which could explain why XAV939 treatment does not reverse the enhanced resistance to gefitinib in PC9 SRPK1 cells (Fig. S6D), suggesting that SRPK1 regulates β-catenin ubiquitination in response to XAV939 treatment. We found that the spacer domain of SRPK1 promoted β-catenin nuclear accumulation (Fig. S6E). These results indicated that the SRPK1-GSK3β axis activated the Wnt/β-catenin pathway in NSCLC cells.

Real-time PCR revealed that *EGFR* mRNA levels were increased in SRPK1-overexpressing cells, and decreased in SRPK1 knockdown cells (Fig. 6B). Similarly, we found that upregulation of SRPK1 increased the expression and phosphorylation of membrane EGFR (mEGFR) (Fig. 6C). We also found that the SRPK1 spacer domain promoted EGFR expression (Fig. S6F). Next, we knocked down *LEF1* to investigate its functions in the Wnt pathway. The mRNA and protein levels of mEGFR were similarly downregulated after LEF1 depletion in both SRPK1-overexpressing NCI-H1650 and NCI-H1975 cell lines (Fig. 6D, E). Furthermore, LEF1 depletion significantly decreased the IC_50_ of gefitinib, indicating that inhibition of the Wnt/β-catenin pathway in SRPK1-overexpressing cells restored gefitinib sensitivity (Fig. 6F). Thus, these results suggested that SRPK1 overexpression drives EGFR signaling and gefitinib resistance via activation of the Wnt/β-catenin pathway, and that gefitinib sensitivity can be restored by LEF1 knockdown.

To study the regulatory mechanism of β-catenin nuclear translocation following increased EGFR expression, we predicted three LEF1 binding sites in a 2-kb EGFR promoter region using the JASPAR website (Fig. 6G). Next, we generated luciferase reporter plasmids carrying wild-type or mutant promoter regions (Fig. 6H) and observed that both mutants had significantly reduced EGFR transcriptional activity in luciferase reporter assays (Fig. 6I). Moreover, we investigated whether the transcriptional activity of mutants is regulated by Wnt3a. The deletion of region#1 reduced the EGFR transcriptional activity with or without Wnt3a treatment, whereas Wnt3a restored the decrease induced by deletion of region#2, suggesting that region#1 is the crucial site for LEF1 binding to the EGFR promoter region (Fig. 6I). ChIP-qPCR assays showed that SRPK1 depletion significantly reduced the enrichment of region#1 by β-catenin and LEF1 (Fig. 6J and Fig. S6G). In accordance with this result, LEF1 depletion significantly reduced the enrichment of region#1 by β-catenin in both SRPK1-overexpressing NCI-H1650 and NCI-H1975 cell lines (Fig. S6H and S6I). This indicated that SRPK1 is a crucial factor in promoting the binding between β-catenin and the EGFR promoter region. Taken together, our results indicated that the non-kinase domain of SRPK1, a positive regulator directly affecting Wnt/β-catenin pathway, facilitates the regulation of mEGFR levels and tumor growth under gefitinib treatment in NSCLC.

### SRPK1 expression is a prognostic factor associated with EGFR expression in NSCLC patients

**Fig. 7:**
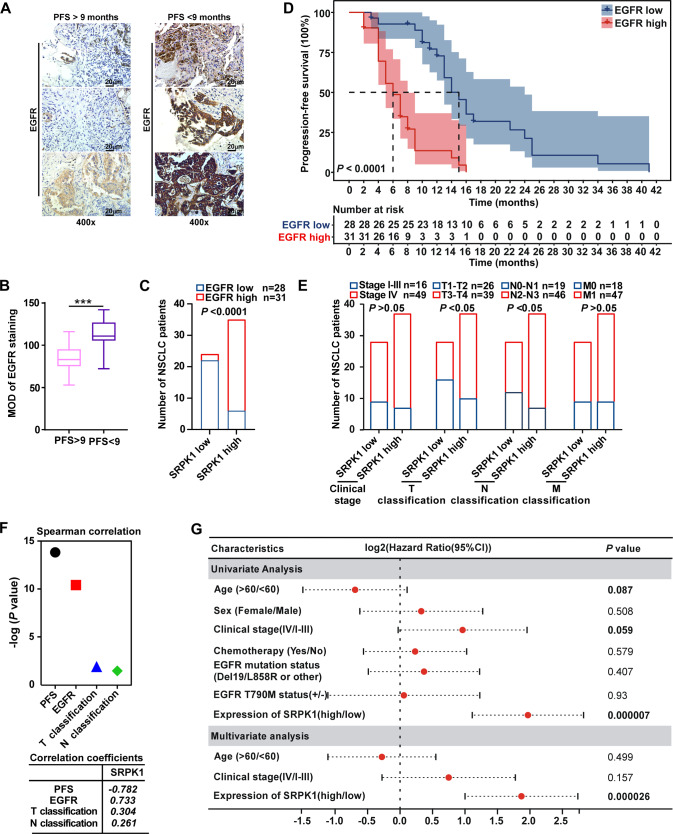
SRPK1 expression is a prognostic factor associated with EGFR expression in NSCLC patients. **A** Representative images of IHC staining of EGFR in sections from NSCLC patients (*n* = 59); scale bars: 20 μm. **B** Quantification of EGFR IHC staining; data represent the mean ± SD and whiskers represent 5 and 95 percentiles. **C** Correlation between SRPK1 and EGFR (Pearson’s chi-squared (χ^2^) test; the number of NSCLC patients is shown). **D** Kaplan-Meier curves used to estimate NSCLC patient PFS in the high- or low-EGFR expression groups (Log-rank test). **E** Clinical stage and TNM classifications of high- and low- SRPK1 expression groups (Pearson’s chi-squared (χ^2^) test; the number of NSCLC patients is shown). **F** Spearman rank analysis used to verify the correlation between SRPK1 and PFS, T classification or EGFR. The correlation coefficients are shown in the panel below. **G** Forest plots showing the association of characteristics with PFS in NSCLC cases. The significant variables (*P* < 0.1) in univariate analysis were analyzed using multivariate regression. HR > 1 indicates increased risk (decreased survival), and HR < 1 indicates decreased risk (increased survival). *P < 0.05, **P < 0.01 and ***P < 0.001.

Our results revealed that SRPK1 overexpression limited the therapeutic response to EGFR-TKI by promoting membrane EGFR expression, which was associated with poor PFS in clinical samples (Fig. 1). Therefore, we then analyzed the relationship between SRPK1 and EGFR expression levels in tissues from 59 NSCLC patients. IHC analysis revealed that total EGFR (tEGFR) was highly expressed in the PFS ≤ 9 group (Fig. 7A, B), which was consistent with SRPK1 expression. Chi-squared (χ^2^) tests further revealed that SRPK1 expression was positively correlated with tEGFR expression (Fig. 7C). Kaplan-Meier analysis also demonstrated that high tEGFR expression was associated with poor PFS following EGFR-TKI therapy (Fig. 7D). Chi-squared (χ^2^) test revealed that T classification was also significantly associated with SRPK1 expression in advanced NSCLC patients treated with EGFR-TKI (Fig. 7E). Spearman correlation confirmed the relationships between SRPK1 and PFS, T classification or tEGFR expression (Fig. 7F). Additionally, multivariate regression analysis indicated that SRPK1 expression was a prognostic factor independent of resistance mutations, increasing the risk of progression following EGFR-TKI therapy (Fig. 7G). Taken together, our findings indicated that SRPK1 overexpression increased tEGFR expression and shortened the time interval for disease progression in advanced NSCLC patients receiving TKI therapy, resulting in a poor prognosis.

To investigate the function of β-catenin in EGFR-TKI resistance induced by SRPK1, we also analyzed the expression of β-catenin in 54 NSCLC patients. However, there was no significant difference in the total β-catenin protein levels between the PFS ≤ 9 group and PFS > 9 groups (Fig. S7A). Chi-squared (χ^2^) tests revealed that there’s no correlation between β-catenin and SRPK1 expression (Fig. S7B). Kaplan-Meier analysis also demonstrated that the total β-catenin protein level was not significantly correlated with PFS during EGFR-TKI therapy (Fig. S7C). Thus, these results suggested that SRPK1-mediated Wnt activation and membrane EGFR expression is induced by nuclear accumulation of β-catenin rather than the total β-catenin protein level.

## Discussion

The mechanisms underlying the acquisition of gefitinib resistance primarily include secondary mutations within *EGFR*, activation of parallel receptor tyrosine kinases and upregulation of EGFR effector proteins [29–31]. However, the intrinsic mechanisms underlying EGFR-TKI resistance remain unclear, and further studies are urgently required. Aberrant activation of signaling pathways in NSCLC cells has been reported to play a vital role in the development of EGFR-TKI resistance [29–31]. Given the considerable heterogeneity of patients with acquired drug resistance and the fact that EGFR-TKI resistance is seemingly inevitable [32], we aimed to explore the key kinases that participate in intrinsic EGFR-TKI resistance to identify potential novel targets to counteract this process. SRPK1 has been identified as a critical downstream protein in the EGFR pathway [23], and its constitutive activation promotes SRPK1 translocation into the nucleus, where it affects splicing [23, 24]. However, the oncogenic functions of SRPK1 in EGFR-TKI resistance have not yet been fully characterized. In the present study, SRPK1 was found to be highly expressed in gefitinib-resistant cells, and high SRPK1 expression was independently associated with poor prognosis in 65 NSCLC patients who underwent EGFR-TKI therapy, indicating that SRPK1 plays an important role in EGFR-TKI resistance.

High EGFR expression is positively associated with cancer progression [33]. EGFR signaling is preceded by receptor dimerization and, importantly, EGFR overexpression is known to increase unbound homodimer levels, which has been proposed as a mechanism of spurious kinase activation in the absence of ligands [34, 35]. In the present study, we focused on the molecular mechanism by which EGFR overexpression leads to EGFR-TKI resistance. Our study demonstrated that upregulated SRPK1 expression limits gefitinib-induced apoptosis and promotes overexpression and hyperactivation of mEGFR. Retrospective IHC analysis of EGFR in tissue samples from patients confirmed a correlation between SRPK1 and EGFR expression. These results strongly suggested that SRPK1 promotes EGFR-TKI resistance in NSCLC by escaping apoptosis during the initial treatment and subsequently, increasing receptor levels to accelerate progression.

The Wnt and EGFR signaling pathways play a role in both embryonic development and cell proliferation, and abnormal activation of both pathways can lead to tumorigenesis [36, 37]. Aberrant activation of Wnt/β-catenin signaling is also involved in drug resistance in a range of cancers [38–40]. Cross-talk between the Wnt/β-catenin and EGFR signaling pathways has been reported, in which the binding of Wnt ligands to receptors activates the EGFR signaling pathway, and EGFR activate β-catenin via the receptor tyrosine kinase-PI3K/Akt pathway [41]. Previous studies have shown that shRNA-mediated inhibition of the Wnt/β-catenin pathway significantly increases the efficacy of EGFR-TKI both in vivo and in vitro [28]. In the present study, we observed that SRPK1 upregulation increased the expression and phosphorylation of mEGFR in gefitinib-sensitive cells, whereas LEF1 knockdown suppressed these processes, reversing TKI resistance. ChIP-qPCR and luciferase assays further demonstrated that SRPK1 promotes the binding between β-catenin/LEF1 and the EGFR promoter region to activate transcription. We also confirmed that SRPK1 promotes nuclear accumulation of β-catenin to activate the Wnt pathway, although this was not confirmed in clinical samples due to technical problems. Thus, our study demonstrated that SRPK1 is a critical regulator of multiple pathways, linking upstream signaling from EGFR and Wnt/β-catenin to downstream effectors such as AKT.

GSK3β is a key node of the Wnt/β-catenin, EGFR, and Ras/Raf/MEK/ERK signaling pathways and is associated with tumorigenesis [42]. GSK3β phosphorylation at Ser9, a kinase inhibitory site, is a prerequisite for β-catenin phosphorylation to activate Wnt/β-catenin pathway. In the present study, we found that GSK3β is involved in gefitinib resistance in a dose-dependent manner and that SRPK1 promotes GSK3β autophosphorylation at Ser9. GSK3β is also a critical target of the PI3K/AKT pathway, with GSK3β Ser9 also being phosphorylated by activated AKT [43]. Aberrant SRPK1 expression is known to induce constitutive AKT activation [23]. However, AKT inhibitors failed to block GSK3β phosphorylation at Ser9 in SRPK1-overexpressing cell lines in the present study, suggesting that SRPK1 contributes to gefitinib resistance independently of PI3K/AKT signaling. The modulation activity of SRPK1 was also associated with its transient interactions with non-shuttling protein complexes, such as scaffold attachment factors (SAF; SAFB1 and SAFB2). The catalytic activity of SRPK1 is compromised by its interaction with SAFBs [44]. In the present study, we demonstrated that the non-kinase domain of SRPK1 mediates its interaction with GSK3β and, ultimately, increases resistance to gefitinib in NSCLC. ADP release controls protein kinase A (PKA) catalysis [45], and our results suggested that the SRPK1 spacer domain-GSK3β interaction increases ADP release, which is a rate-limiting process. Taken together, these findings suggest that SRPK1 acts as a scaffold protein to promote gefitinib resistance. As the conjugation site for SRPK1-GSK3β binding, the spacer domain may serve as a target for specifically designed short-peptide inhibitors to reverse gefitinib resistance in patients with NSCLC.

Recently, signal transducer and activator of transcription 3 (STAT3) and Src-YES-associated protein 1 (YAP1) signaling have been reported to be activated during EGFR-TKI treatment and to limit the therapeutic response [46]. YAP signaling regulates transcriptional reprogramming of the apoptotic Hippo pathway, directly targeting SLUG to inhibit Bcl-2-modifying-factor (BMF), which limits EGFR-TKI-induced apoptosis [47]. Nuclear YAP also acts as a regulator of the Wnt/β-catenin pathway, which upregulates β-catenin expression [48]. In addition, YAP is known to regulate the expression of the tumor suppressor PTEN and influence the PI3K–mTOR pathway. High SRPK1 expression and negative PTEN have a synergistic effect on adverse clinical outcomes in prostate cancer [49]. Therefore, the role of SRPK1 as a linker in multiple pathways warrants further exploration.

In conclusion, we demonstrated that the SRPK1 spacer domain, but not the SRPK1 kinase activity, is required for EGFR-TKI intrinsic resistance. Our results suggest that SRPK1 increases the accumulation and phosphorylation of membrane EGFR via its spacer domain, which binds directly to GSK3β to enhance its autophosphorylation at Ser9. Additional investigations concerning the spacer domain of SRPK1 will improve our understanding of tumor progression and help to determine whether SRPK1 represents a viable therapeutic target in gefitinib-resistant NSCLC patients. Our study provides novel insights into EGFR-TKI resistance by revealing the important role of SRPK1 spacer domain in limiting the response to gefitinib by inhibiting apoptosis.

## Materials and methods

### Cell culture

NCI-H1650 (CRL-5883; ATCC, USA), NCI-H1975 (CRL-5908; ATCC), PC9, PC9GR (gefitinib-resistant PC9) (Shanghai Ruilu Biotechnology Co., Ltd., Shanghai, China) and 293 T cells (CRL-3216, ATCC) were cultured in Dulbecco’s modified Eagle’s medium (DMEM; Gibco, MA, USA) supplemented with 10% fetal bovine serum (FBS; Gibco, USA) and 1% penicillin/streptomycin (Gibco, USA) at 37 °C under 5% CO_2_. Adherent cells were released for passaging by treatment with trypsin (0.25%, Gibco, USA). The gefitinib-resistant cell line PC9GR was established by continuous culture in the presence of an increasing concentration of gefitinib, starting from half of the IC_50_ concentration of the sensitive parent line (PC9). Sensitive cells gradually die during the culture period. Between each increase in the concentration of gefitinib, the culture medium was discarded and the cells washed three times with phosphate-buffered saline (PBS). This process was repeated until the cell line exhibited stable growth at 100-fold IC_50_ of gefitinib over a period of between 6 and 18 months. The short tandem repeat (STR) profiles of all the cell lines are shown in Supplementary Table 2. The presence of the T790M mutation in PC9GR was confirmed by first-generation sequencing.

### Patients and tissue specimens

This study included 65 patients diagnosed with NSCLC harboring the EGFR mutation and who received EGFR-TKI therapy at Shenzhen People’s Hospital and Henan Provincial People Hospital between February 2017 and August 2022. Demographic and clinical characteristics at the time of pathological examination were obtained from medical records and confirmed by a pathologist (Supplementary Table 1). PFS was defined as the time from the first date of EGFR-TKI treatment to the point of progression, initiation of the next treatment, death, or last follow-up.

### Tumor xenograft model

Forty male BALB/c nude mice (aged 5–6 weeks, 16–18 g) were purchased from Beijing Vital River Laboratory Animal Technology Co. Ltd. PC9, PC9GR, PC9-vector, PC9-SRPK1, PC9GR-vector, and PC9GR-SRPK1-sh2# cells (1×10^6^) were mixed with Matrigel and injected subcutaneously into the inguinal folds of the mice. Tumor volumes were measured every three days, and when tumor volumes reached 70–100 mm^3^ the mice were divided randomly into the indicated groups and intraperitoneally injected with 100 μL DMSO or 5 mg/kg gefitinib every three days. After 27 days, the mice were sacrificed by carbon dioxide asphyxiation and the tumors were dissected.

### Immunohistochemistry (IHC)

Paraffin-embedded pathological tissue sections from NSCLC patients and excised tumors from xenograft mice were labeled with antibodies as previously described [25]. All details of antibodies used in IHC are provided in Supplementary Table 3. The mean of optical density (MOD) was determined using Image J software. For patient sections, the percentage of positive cells and staining intensity in the tumor cells were assessed and quantitatively scored by a pathologist. The intensity of staining was classified on a scale of 0 to 3: 0, negative; 1, weak; 2, moderate; and 3, strong. The IHC score was calculated as a percentage (%) × intensity, with a range of 0 to 3.

### Western blot analysis

Western blot analysis was performed as previously described [25]. In brief, the cells were washed by cold PBS for three times and added lysis buffer (50 mM sodium chloride, 1.0% Triton X-100 and 50 mM Tris pH 8.0) to extract the proteins. The protein concentration was measured using a BCA protein assay kit (Pierce, Rockford, IL). In total, 20 μg of protein were separated by sodium dodecyl sulfate-polyacrylamide gel (5–15%) electrophoresis and transferred electrophoretically onto a PVDF membrane (Amersham, Little Chalfont, UK). Membranes were blocked in 5% non-fat dried milk in PBST (0.1% Tween 20) and incubated overnight at 4 °C with primary detection antibodies diluted with PBS containing 1% BSA. Details of the primary detection antibodies are listed in Supplementary Table 3. Membranes were washed in PBST for 20 min and incubated at room temperature for 2 h with horseradish peroxidase-conjugated anti-rabbit IgG or anti-mouse IgG (1:10,000, CST) secondary antibody diluted with PBST. Total and phosphorylated protein levels were quantified by densitometric scanning using Image J software.

### Immunofluorescence assay

Immunofluorescence assays were performed as described previously [25]. Briefly, the cells were incubated with anti-β-catenin (1:100) antibody overnight at 4 °C, followed by incubation with secondary antibodies at 37 °C for 1 h. The cells were visualized using a Dragonfly laser scanning confocal microscopy system (Andor, UK).

### Real-time quantitative PCR (qRT-PCR) analysis

Total RNA was extracted from NSCLC cells using RNAiso Plus (TaKaRa, Shiga, Japan) according to the manufacturer’s instructions. cDNA was synthesized from total RNA using random primers, and real-time PCR was performed using a Real-Time system (CFX96, Bio-Rad Laboratories, Inc., CA, USA) [19]. Each reaction was carried out in a 96-well optical grade PCR plate, sealed with optical sealing tape. Amplifications were carried out in a total reaction volume of 20 μL containing 2× SYBR Green qPCR Master Mix (Bimake), 100 ng cDNA, and 250 nM of each primer using the following reaction parameters: 95 °C for 5 min followed by 35 cycles at 95 °C for 10 s, and 60 °C for 1 min. The expression data were normalized to the geometric mean of the expression of the housekeeping gene *GAPDH* and calculated using the 2^−ΔΔCq^ method [50]. Primer sequences are listed in Supplementary Table 4. β-catenin short hairpin RNA (shRNA) (CATAACCTTTCCCATCATCG) sequence was cloned into pLKO.1-puro vectors to knock-down β-catenin expression. LEF1 shRNA (CCATCAGATGTCAACTCCAAA) sequence was cloned into pLKO.1-puro vectors to knock-down LEF1 expression.

### Plasmids, retroviral infection, and transfection

Human SRPK1 FL (full length), ΔN (no N-terminus), ΔS (no spacer domain), ΔK1 (no kinase domain I-VI), ΔK2 (no kinase domain VII–XI), and GSK3β cDNA sequences were amplified and then cloned into pLVX-mcherry-C_1_ lentiviral vectors or pcDNA3.0 vectors with HA, Flag, His or GST tags. GSK3β (S9A, K85A, and S9A/K85A) and SRPK1 (K109A) mutants were generated using the Mut Express II Fast Mutagenesis kit V2 (cat: C214–01, Vazyme, Nanjing, China). Two human SRPK1-targeting short hairpin RNA (shRNA) (1#: GAACAACACATTAGCCAACTT; 2#: GCTGAAGTCAGTTCGCAATTC) sequences were cloned into pLKO.1-puro vectors to knock-down SRPK1 expression. Stable cell lines were generated by retroviral infection followed by selection using 0.5 µg/mL puromycin for 10 days as previously described [25]. The primer sequences used are listed in Supplementary Table 5. Schematic diagrams of the SRPK1 truncated plasmids are shown in Fig. 5C and S4A.

### Nuclear extract and plasma membrane protein preparation

Cells were washed with 5 mL PBS containing a protease inhibitor cocktail (Bimake, Houston, TX, USA) and a phosphatase inhibitor cocktail (Bimake). The cells were transferred to a pre-chilled 15-ml conical tube and centrifuged at 200 × *g* for 5 min at 4 °C. Nuclear Extract kits (cat: 40010, Active Motif, Rixensart, Belgium) and Plasma membrane protein isolation kits (cat: SM-005, Invent Biotechnologies, Inc., Plymouth, UK) were then used to isolate the nuclear extracts and plasma membrane protein from the cell pellets according to the manufacturers’ protocols.

### MTT cytotoxicity assay

Cytotoxicity assays were performed as described previously [51]. Cells (5 × 10^3^ cells/well) were plated in 96-well plates and treated with the indicated concentrations of gefitinib for 48 h. Twenty microliters of MTT reagent (5 mg/mL) were then added to each well (96-well plate) and incubated for 4 h. Cell viability was assessed by measuring the absorbance at 570 nm. Half-maximal inhibitory concentration (IC_50_) values of gefitinib were calculated using GraphPad Prism (version 7) by nonlinear regression (curve fit).

### Annexin V/propidium iodide (PI) staining

The cells were collected, washed twice with PBS, and resuspended in binding buffer at 1 × 10^6^ cells/mL. These cells were incubated with 5 µL Annexin V-FITC (cat: 556420; Biosciences, USA) and 5 µL PI for 15 min at room temperature in the dark. After adding 400 µL binding buffer, the samples were kept on ice and analyzed using a BD FACSCalibur flow cytometer (NJ, USA) within 1 h. The data were analyzed using FlowJo software version 10 (FlowJo LLC., USA).

### Terminal deoxynucleotidyl transferase nick-end-labeling (TUNEL) assay

Cells (5 × 10^4^) on coverslips were treated with the indicated concentrations of gefitinib for 48 h, fixed with 4% paraformaldehyde for 25 min and washed twice with PBS. After further washing with PBS, the cells were incubated on ice with 0.1% Triton X-100 in 0.1% sodium citrate for 2 min. The cells were then washed with PBS and incubated with 50 µL TUNEL reaction mixture (cat: G3250; Promega, USA) containing the rTdT enzyme for 1 h at 37°C. After washing three times with PBS, the cells were incubated with 50 µL PI for 15 min in the dark, washed, and analyzed under an inverted fluorescence microscope (CKX53; Olympus, Japan). TUNEL-positive (apoptotic) cells were quantified by counting green-colored cells in 10 fields.

### Luciferase reporter assay

Luciferase reporter assays were performed as previously described [51]. Briefly, cells (2 × 10^5^) were seeded into 24-well plates and then transiently transfected with TOP FLASH or FOP FLASH and Renilla pRL-TK plasmids. (cat: E2231; Promega) using Lipofectamine 3000 Reagent (cat: 11668019, Thermo Fisher Scientific). After 48 h, relative luciferase activity was measured using a dual-luciferase reporter assay detection kit (cat: E1980; Promega). Briefly, the culture medium was discarded, and the cells were washed with PBS and lysed with passive lysis buffer (Promega). The cell lysates were transferred to a 96-well plate luminometer (Corning, New York, USA) and analyzed using a microplate reader (Bioteck, Winooski, VT, USA). Calculated the ratio of firefly luciferase luminescence (TOP FLASH or FOP FLASH) to Renilla luciferase luminescence for each sample, and then normalized the ratio of experimental sample wells to the ratio of control sample wells

### Chromatin immunoprecipitation assay (ChIP)-qPCR

ChIP assays were performed using the method described by Schmidt with some modifications [52]. Briefly, chromatin and protein complexes were cross-linked using 1.42% formaldehyde and lysed. The soluble supernatant was sonicated and pipetted 50 μL as input, and the remainder was incubated with β-catenin or mouse IgG and protein G-agarose beads overnight at 4 °C. The DNA was purified, recovered after elution and de-crosslinking and analyzed by qPCR.

### Kinase activity assay

HA-tagged or His-tagged SRPK1 was then incubated with glutathione-S-transferase (GST)-tagged GSK3β in kinase assay buffer (25 mM Tris, pH 7.5, 10 mM MgCl2, 2 mM DTT, 5 mM β-Glycerolphosphate, 0.1 mM Na3VO4, and 2 mM EGTA, 20 μM ATP) for 30 min at 30 °C. For isotope radiation kinase assays, His-tagged SRPK1 was incubated with GST-tagged GSK3β in kinase assay buffer containing 1 µL Ci [γ-^32^P] ATP for 30 min at 30°C. The samples were analyzed by SDS-PAGE and autoradiography.

### Co-immunoprecipitation (Co-IP) assay

Cells were cultured in 100-mm culture dishes and lysed by incubation in 500 μL IP lysis buffer (25 mM HEPES [pH 7.4], 150 mM NaCl, 1% NP-40, 1 mM EDTA, 2% glycerol, 1 mM phenylmethylsulfonyl fluoride) on ice for 30 min. The lysates were clarified by microcentrifugation at 14,000 × *g* for 10 min and precleared by incubation with 20 μL agarose beads (cat: 20421, Thermo, USA) for 1 h under rotation at 4 °C. Following centrifugation at 2,000 × *g* for 1 min, the supernatants were incubated overnight at 4 °C with 20 µL anti-SRPK1 (cat: 611072, BD) or anti-GSK3β (cat: 32391, Abcam) antibody-cross-linked protein A/G-agarose beads. The agarose beads were then washed six times with wash buffer (25 mM HEPES [pH 7.4], 150 mM NaCl, 0.5% NP-40, 1 mM EDTA, 2% glycerol, 1 mM PMSF). All liquid was removed, and the pelleted beads were then resuspended in 30 μL lysis buffer for Western blotting using the indicated antibodies.

For IP-MASS assays, 293 T cell were transiently transfected with HA-vector or HA-SRPK1 plasmids. After 48 h, the cells were lysed with IP lysis buffer, and the supernatants were collected by centrifugation at 12,000 × *g* for 10 min at and incubated overnight at 4 °C with 20 μL anti-HA antibody-cross-linked protein G-agarose beads (Sigma). Subsequently, the beads were washed six times with lysis buffer and IP-MASS analysis was performed by Shenzhen Weinafei Biotechnology Co., Ltd. All the SRPK1 interactors identified by IP-MASS in 293 T cells were listed in Supplemental Table 6

### GST-pulldown assay

His-tagged proteins were incubated with bead-bound GST fusion proteins or anti-GST antibody beads (17–0756-01, GE Healthcare) only. The beads were washed at least five times with PBS and protein interactions were determined by Western blotting using the indicated antibodies.

### Bioinformatics analysis

The microarray data obtained in this study were deposited in the Gene Expression Omnibus database (NCBI/GEO/GSE 14925, NCBI/GEO/GSE 47206, and NCBI/ GEO/GSE 34228). The NSCLC dataset (GSE75309) was evaluated by Gene Set Enrichment Analysis (GSEA). *Enrichr* (https://maayanlab.cloud/Enrichr/) was used to identify enriched differential pathways in the database (GSE129221) and relevant pathways among the proteins interacting with SRPK1 identified by IP-MASS. *JASPAR* was used to predict the binding sites of LEF1 in the EGFR promoter region (http://jaspar.genereg.net/).

### Statistical analysis

All statistical analyses were performed using SPSS (version 26.0), GraphPad Prism (version 7) and R (version 3.6.3). Differences between two groups were calculated using Student’s *t* test or the Mann–Whitney U-test according to the data distribution (normal or non-normal, respectively). The IHC scores of SRPK1, EGFR, and β-catenin in patients were categorized into high-value and low-value groups based on the optimal cut-off values derived from the ROC curve analysis. Pearson’s chi-squared (χ^2^) test was used to compare categorical variables of pathological characteristics between different groups. Kaplan-Meier analysis was used to estimate the survival curves for PFS in the two groups using the log-rank test. Spearman rank correlation coefficients were used to estimate the correlations between two variables. Univariable and multivariable Cox proportional hazards models were used to assess the performance of SRPK1 in predicting prognosis. The hazard ratios (HR) with 95% confidence intervals (CI) were based on PFS. A two-sided *P*-value <0.05 was considered to indicate statistical significance.

### Study approval

All patients provided written informed consent to participate in this study. Ethical approval for use in this study were obtained from the Institutional Research Ethics Committee of Shenzhen People’s Hospital (approval no. 2019020). All animal procedures were approved by the Peking University Laboratory Animal Center of Shenzhen Graduate School (approval no. 10837) and complied with the relevant ethical regulations.

## Supplementary information

Supplemental Figure 1

Supplemental Figure 2

Supplemental Figure 3

Supplemental Figure 4

Supplemental Figure 5

Supplemental Figure 6

Supplemental Figure 7

Supplemental figure legends

Table S1

Table S2

Table S3

Table S4

Table S5

Table S6

## Data Availability

All data generated or analyzed during this study are included in this published article and its supplementary information files or are available from the corresponding author upon reasonable request.

## References

[1] Jemal A, Siegel R, Ward E, Murray T, Xu J, Thun MJ (2007). Cancer statistics, 2007. CA Cancer J Clin.

[2] Siegel RL, Miller KD, Fuchs HE, Jemal A (2021). Cancer statistics, 2021. CA Cancer J Clin.

[3] Devarakonda S, Morgensztern D, Govindan R (2015). Genomic alterations in lung adenocarcinoma. Lancet Oncol.

[4] Sharma SV, Bell DW, Settleman J, Haber DA (2007). Epidermal growth factor receptor mutations in lung cancer. Nat Rev Cancer.

[5] Uzman A (2008). The biology of cancer by R. A. Weinberg. Biochem Mol Biol Edu.

[6] Hirsh V (2011). Afatinib (BIBW 2992) development in non-small-cell lung cancer. Future Oncol (Lond, Engl).

[7] Uribe ML, Marrocco I, Yarden Y (2021). EGFR in cancer: signaling mechanisms, drugs, and acquired resistance. Cancers (Basel).

[8] Suda K, Mitsudomi T, Shintani Y, Okami J, Ito H, Ohtsuka T (2021). Clinical impacts of EGFR mutation status: analysis of 5780 surgically resected lung cancer cases. Ann Thorac Surg.

[9] Sequist LV, Waltman BA, Dias-Santagata D, Digumarthy S, Turke AB, Fidias P (2011). Genotypic and histological evolution of lung cancers acquiring resistance to EGFR inhibitors. Sci Transl Med.

[10] Yano S, Wang W, Li Q, Matsumoto K, Sakurama H, Nakamura T (2008). Hepatocyte growth factor induces gefitinib resistance of lung adenocarcinoma with epidermal growth factor receptor-activating mutations. Cancer Res.

[11] Tumbrink HL, Heimsoeth A, Sos ML (2021). The next tier of EGFR resistance mutations in lung cancer. Oncogene.

[12] Fukuoka M, Yano S, Giaccone G, Tamura T, Nakagawa K, Douillard JY (2003). Multi-institutional randomized phase II trial of gefitinib for previously treated patients with advanced non-small-cell lung cancer (The IDEAL 1 Trial) [corrected]. J Clin Oncol.

[13] Schoenfeld AJ, Chan JM, Kubota D, Sato H, Rizvi H, Daneshbod Y (2020). Tumor analyses reveal squamous transformation and off-target alterations as early resistance mechanisms to first-line osimertinib in EGFR-mutant lung cancer. Clin Cancer Res.

[14] Jotte RM, Spigel DR (2015). Advances in molecular-based personalized non-small-cell lung cancer therapy: targeting epidermal growth factor receptor and mechanisms of resistance. Cancer Med.

[15] Cortot AB, Janne PA (2014). Molecular mechanisms of resistance in epidermal growth factor receptor-mutant lung adenocarcinomas. Eur Respir Rev.

[16] Dong JK, Lei HM, Liang Q, Tang YB, Zhou Y, Wang Y (2018). Overcoming erlotinib resistance in EGFR mutation-positive lung adenocarcinomas through repression of phosphoglycerate dehydrogenase. Theranostics.

[17] Zhou B, Li YD, Deng Q, Wang HX, Wang YP, Cai B (2013). SRPK1 contributes to malignancy of hepatocellular carcinoma through a possible mechanism involving PI3K/Akt. Mol Cell Biochem.

[18] Giannakouros T, Nikolakaki E, Mylonis I, Georgatsou E (2011). Serine-arginine protein kinases: a small protein kinase family with a large cellular presence. FEBS J.

[19] Huang JQ, Li HF, Zhu J, Song JW, Zhang XB, Gong P (2021). SRPK1/AKT axis promotes oxaliplatin-induced anti-apoptosis via NF-kappaB activation in colon cancer. J Transl Med.

[20] Tzelepis K, De Braekeleer E, Aspris D, Barbieri I, Vijayabaskar MS, Liu WH (2018). SRPK1 maintains acute myeloid leukemia through effects on isoform usage of epigenetic regulators including BRD4. Nat Commun.

[21] Gou L-T, Lim D-H, Ma W, Aubol BE, Hao Y, Wang X (2020). Initiation of parental genome reprogramming in fertilized oocyte by splicing kinase SRPK1-catalyzed protamine phosphorylation. Cell.

[22] Amin EM, Oltean S, Hua J, Gammons MVR, Hamdollah-Zadeh M, Welsh GI (2011). WT1 mutants reveal SRPK1 to be a downstream angiogenesis target by altering VEGF splicing. Cancer Cell.

[23] Wang P, Zhou Z, Hu A, Albuquerque CPD, Fu XD (2014). Both decreased and increased SRPK1 levels promote cancer by interfering with PHLPP-mediated dephosphorylation of Akt. Mol Cell.

[24] Zhou Z, Qiu J, Liu W, Zhou Y, Plocinik RM, Li H (2012). The Akt-SRPK-SR axis constitutes a major pathway in transducing EGF signaling to regulate alternative splicing in the nucleus. Mol Cell.

[25] Gong LY, Song JW, Lin X, Wei FK, Zhang CC, Wang ZM (2016). Serine-arginine protein kinase 1 promotes a cancer stem cell-like phenotype through activation of Wnt/beta-catenin signalling in NSCLC. J Pathol.

[26] Tan X, Apte U, Micsenyi A, Kotsagrelos E, Luo JH, Ranganathan S (2005). Epidermal growth factor receptor: a novel target of the Wnt/beta-catenin pathway in liver. Gastroenterology.

[27] Duchartre Y, Kim YM, Kahn M (2016). The Wnt signaling pathway in cancer. Crit Rev Oncol Hemat.

[28] Casas-Selves M, Kim J, Zhang Z, Helfrich BA, Gao D, Porter CC (2012). Tankyrase and the canonical Wnt pathway protect lung cancer cells from EGFR inhibition. Cancer Res.

[29] Bean J, Brennan C, Shih JY, Riely G, Viale A, Wang L (2007). MET amplification occurs with or without T790M mutations in EGFR mutant lung tumors with acquired resistance to gefitinib or erlotinib. Proc Natl Acad Sci USA.

[30] Takeuchi K, Soda M, Togashi Y, Suzuki R, Sakata S, Hatano S (2012). RET, ROS1 and ALK fusions in lung cancer. Nat Med.

[31] Tan CS, Gilligan D, Pacey S (2015). Treatment approaches for EGFR-inhibitor-resistant patients with non-small-cell lung cancer. Lancet Oncol.

[32] Leonetti A, Sharma S, Minari R, Perego P, Giovannetti E, Tiseo M (2019). Resistance mechanisms to osimertinib in EGFR-mutated non-small cell lung cancer. Br J Cancer.

[33] Talukdar S, Emdad L, Das SK, Fisher PB (2020). EGFR: An essential receptor tyrosine kinase-regulator of cancer stem cells. Adv Cancer Res.

[34] Caldieri G, Malabarba MG, Di Fiore PP, Sigismund S (2018). EGFR trafficking in physiology and cancer. Prog Mol Subcell Biol.

[35] Chung I, Akita R, Vandlen R, Toomre D, Schlessinger J, Mellman I (2010). Spatial control of EGF receptor activation by reversible dimerization on living cells. Nature.

[36] Parsons MJ, Tammela T, Dow LE (2021). WNT as a driver and dependency in cancer. Cancer Disco.

[37] Friedlaender A, Subbiah V, Russo A, Banna GL, Malapelle U, Rolfo C (2022). EGFR and HER2 exon 20 insertions in solid tumours: from biology to treatment. Nat Rev Clin Oncol.

[38] He LF, Zhu H, Zhou SY, Wu T, Wu H, Yang H (2018). Wnt pathway is involved in 5-FU drug resistance of colorectal cancer cells. Exp Mol Med.

[39] Kerdidani D, Chouvardas P, Arjo AR, Giopanou I, Ntaliarda G, Guo YA (2019). Wnt1 silences chemokine genes in dendritic cells and induces adaptive immune resistance in lung adenocarcinoma. Nat Commun.

[40] Wickstrom M, Dyberg C, Milosevic J, Einvik C, Calero R, Sveinbjornsson B (2015). Wnt/beta-catenin pathway regulates MGMT gene expression in cancer and inhibition of Wnt signalling prevents chemoresistance. Nat Commun.

[41] Hu T, Li C (2010). Convergence between Wnt-β-catenin and EGFR signaling in cancer. Mol Cancer.

[42] McCubrey JA, Steelman LS, Bertrand FE, Davis NM, Abrams SL, Montalto G (2014). Multifaceted roles of GSK-3 and Wnt/beta-catenin in hematopoiesis and leukemogenesis: opportunities for therapeutic intervention. Leukemia.

[43] Doble BW, Woodgett JR (2003). GSK-3: tricks of the trade for a multi-tasking kinase. J Cell Sci.

[44] Harper SJ, Bates DO (2008). VEGF-A splicing: the key to anti-angiogenic therapeutics?. Nat Rev Cancer.

[45] Zhou J, Adams JA (1997). Participation of ADP dissociation in the rate-determining step in cAMP-dependent protein kinase. Biochemistry.

[46] Chaib I, Karachaliou N, Pilotto S, Codony Servat J, Cai X, Li X (2017). Co-activation of STAT3 and YES-associated protein 1 (YAP1) pathway in EGFR-mutant NSCLC. J Natl Cancer Inst.

[47] Kurppa KJ, Liu Y, To C, Zhang T, Fan M, Vajdi A (2020). Treatment-induced tumor dormancy through YAP-mediated transcriptional reprogramming of the apoptotic pathway. Cancer Cell.

[48] Jiang L, Li J, Zhang C, Shang Y, Lin J (2020). YAPmediated crosstalk between the Wnt and Hippo signaling pathways (Review). Mol Med Rep..

[49] Abou-Ouf H, Assem H, Ghosh S, Karnes RJ, Stoletov K, Palanisamy N (2021). High serine-arginine protein kinase 1 expression with PTEN loss defines aggressive phenotype of prostate cancer associated with lethal outcome and decreased overall survival. Eur Urol Open Sci.

[50] Livak KJ, Schmittgen TD (2001). Analysis of relative gene expression data using real-time quantitative PCR and the 2(-Delta Delta C(T)) Method. Methods.

[51] Huang JQ, Wei FK, Xu XL, Ye SX, Song JW, Ding PK (2019). SOX9 drives the epithelial-mesenchymal transition in non-small-cell lung cancer through the Wnt/-catenin pathway. J Transl Med.

[52] Schmidt D, Wilson MD, Spyrou C, Brown GD, Hadfield J, Odom DT (2009). ChIP-seq: Using high-throughput sequencing to discover protein-DNA interactions. Methods.

