# Supplementary material for: Serine-arginine protein kinase 1 (SRPK1) promotes EGFR-TKI resistance by enhancing GSK3β Ser9 autophosphorylation independent of its kinase activity in non-small-cell lung cancer
Source: Oncogene. 2023 Mar 3;42(15):1233–46. doi: 10.1038/s41388-023-02645-2 (PMC10079535; doi:10.1038/s41388-023-02645-2)
Supplement: Supplementary file 8 — Supplemental figure legends [file 41388_2023_2645_MOESM8_ESM.docx]

**Figure S1. SRPK1 inhibits apoptosis in vitro*.*** (A) Flow cytometry and Annexin V FITC/PI staining in the indicated cells treated with increasing concentrations of gefitinib. (B) Representative TUNEL staining images of the indicated cells treated with increasing concentrations of gefitinib.

**Figure S2. SRPK1 promotes gefitinib resistance.** (A) Western blot analysis of SRPK1 levels following gefitinib treatment (0, 1, 5, or 10 µM).

**Figure S3. SRPK1 upregulates GSK3β pSer9 independent of AKT.** (A) Western blot analysis of GSK3β phosphorylation at the basal level in gefitinib resistant cells (H1975, PC9GR) and gefitinib sensitive cells (H1650, PC9). (B) Western blot analysis of GSK3β phosphorylation in NCI-H1975 cell treated or not with gefitinib. (C) Western blot analysis of GSK3β and AKT phosphorylation levels in the indicated cells following exposure to 20 nM triciribine, an AKT inhibitor, for 24 h. (D) Quantification of p-AKT/AKT and p-GSK3β/ GSK3β in (C) levels. **P* < 0.05, ***P* < 0.01 and ****P* < 0.001.

**Figure S4. SRPK1 kinase activity is not required for gefitinib resistance.** (A) Schematic diagram of the SRPK1 fragment domains, and GST pulldown assays performed to identify the sites mediating the SRPK1 and GSK3β protein interaction. (B) Immunofluorescence analysis of SRPK1 sub-cellular localization in the indicated cell lines following exposure to gefitinib. (C) Immunofluorescence analysis of SRPK1 mutant sub-cellular localization in the indicated cell lines. (D) IP analysis of the interactions between SRPK1 and GSK3β in NCI-H1975 cells treated with 0.5, 1 and 2μM SRPINHX31 for 4h. (E) Viability of PC9 cells overexpressing SRPK1 and PC9GR following exposure to increasing concentrations of gefitinib and SRPK1 inhibitors (SRPIN340 and SPHINX31) for 48h; data represent the mean ± SD.

**Figure S5. SRPK1 promotes β-catenin nuclear translocation to activate the Wnt/β-catenin pathway.** (A) GO analysis of differentially expressed genes in PC9 and PC9 GR cells in GSE129221 dataset. (B) GSEA analysis indicating significant correlations between gefitinib treatment and the Wnt pathway (BIOCARTA_WNT_PATHWAY) in the GSE75309 dataset. (C) Luciferase assay of the transcriptional activities of *TCF4/LEF1* in SRPK1-transduced PC9 and NCI-H1650 cells, and in *SRPK1*-silenced PC9GR and NCI-H1975 cells. (D) Immunofluorescence analysis of nuclear β-catenin expression in the indicated cell lines following exposure to increasing concentrations of gefitinib. (E) qRT-PCR analysis of mRNA expression analysis of Wnt/β-catenin pathway downstream genes in the indicated cell lines; data represent the mean ± SD (n = 3). (F) qRT-PCR analysis of mRNA expression analysis of Wnt/β-catenin pathway downstream genes after β-catenin knockdown in the indicated cell lines; data represent the mean ± SD (n = 3). **P* < 0.05, ***P* < 0.01 and ****P* < 0.001.

**Figure S6. The binding between β-catenin and the EGFR promoter region is related on LEF1.** (A) Western blot analysis of total β-catenin levels in SRPK1 overexpressing PC9 cells. (B) Western blot analysis of β-catenin expression levels in the cytoplasmic (C) and nuclear (N) fractions of the indicated cells. (C) Viability of PC9 cells overexpressing SRPK1 following increasing gefitinib exposure and β-catenin inhibitors (XAV-939); data represent the mean ± SD. (D) Western blot analysis of total β-catenin level sin SRPK1 overexpressing NCI-H1650 and PC9 cells exposed to XAV-939. (E) Western blot analysis of β-catenin expression levels in the cytoplasmic (C) and nuclear (N) fractions of the indicated cells following transient transfection with the SRPK1 mutant. Quantification of nuclear β-catenin expression levels is shown in right panel. (F) Western blot analysis of membrane expression level of EGFR in the indicated cells following transient transfection with the SRPK1 mutant. (G) ChIP-qPCR assays with LEF1 antibody and primers across region#1 in SRPK1-silenced or vector-only NCI-H1975 cells. (H) and (I) ChIP-qPCR assays with β-catenin antibody and primers across region#1 in SRPK1 overexpressed or vector-only NCI-H1650 cells and SRPK1-silenced or vector-only NCI-H1975 cells following by LEF1 knockdown. *P < 0.05, **P < 0.01 and ***P < 0.001.

**Figure S7. SRPK1 expression is not correlated with total β-catenin expression in NSCLC patients.** (A) Representative images from IHC staining of β-catenin in sections taken from NSCLC patients (n = 54); scale bars: 20 μm. (B) The relationship between SRPK1 and β-catenin (Pearson’s chi-squared (χ^2^) test; the numbers of NSCLC patients are shown). (C) Kaplan–Meier curves used to estimate NSCLC patient PFS in the high- or low-β-catenin expression groups (Log-rank test).
