# Supplementary material for: Serine-arginine protein kinase 1 (SRPK1) promotes EGFR-TKI resistance by enhancing GSK3β Ser9 autophosphorylation independent of its kinase activity in non-small-cell lung cancer
Source: Oncogene. 2023 Mar 3;42(15):1233–46. doi: 10.1038/s41388-023-02645-2 (PMC10079535; doi:10.1038/s41388-023-02645-2)
Supplement: Supplementary file 9 — Table S1 [file 41388_2023_2645_MOESM9_ESM.docx]

**Table S1: Clinicopathologic characteristics of patient samples and expression of SRPK1 in NSCLC with EGFR-TKIs treatment**

| **Characteristics** | **SRPK1 Low(N=28)** | **SRPK1 High(N=37)** |
| --- | --- | --- |
| **Sex** |  |  |
| Male | 7(25.00%) | 10(27.03%) |
| Female | 21(75.00%) | 27(72.97%) |
| **Age** |  |  |
| ≤60 | 13(46.43%) | 21(56.76%) |
| >60 | 15(53.57%) | 16(43.24%) |
| **Adjuvant chemotherapy** |  |  |
| No | 19(67.86%) | 22(59.46%) |
| Yes | 9(32.14%) | 15(40.54%) |
| **Clinical stage** |  |  |
| I-III | 9(32.14%) | 7(18.92%) |
| IV | 19(67.86%) | 30(81.08%) |
| **T classification** |  |  |
| T1-T2 | 16(57.14%) | 10(27.03%) |
| T3-T4 | 12(42.86%) | 27(72.97%) |
| **N classification** |  |  |
| N0-N1 | 12(42.86%) | 7(18.92%) |
| N2-N3 | 16(57.14%) | 30(81.08%) |
| **M classification** |  |  |
| M0 | 9(32.14%) | 9(24.32%) |
| M1 | 19(67.86%) | 28(75.68%) |
| **Progression-free survival（months）** | |  |
| Mean±SD | 16.57±9.23 | 6.43±4.48 |
| Median[min-max] | 14.00[2.00,41.00] | 5.00[2.00,25.00] |
| **EGFR mutation status, n (%)** |  |  |
| *Del19* | 13(46.43%) | 14(37.84%) |
| *L858R* | 9(32.14%) | 11(29.73%) |
| *Del19&T790M* | 1(3.57%) | 5(13.51%) |
| *L858R&T790M* | 3(10.71%) | 5(13.51%) |
| *P741H +T790M* | 1(3.57%) | 0(0.0%) |
| *Others** | 1(3.57%) | 2(5.41%) |
| ***** Others: G719C*&*S768I、G719S*&*L861QL. | | |
