# Supplementary material for: Serine-arginine protein kinase 1 (SRPK1) promotes EGFR-TKI resistance by enhancing GSK3β Ser9 autophosphorylation independent of its kinase activity in non-small-cell lung cancer
Source: Oncogene. 2023 Mar 3;42(15):1233–46. doi: 10.1038/s41388-023-02645-2 (PMC10079535; doi:10.1038/s41388-023-02645-2)
Supplement: Supplementary file 10 — Table S2 [file 41388_2023_2645_MOESM10_ESM.docx]

**Table S2: STR profiles of cell lines**

| Cell lines  STR loci | NCI-H1650 | NCI-H1975 | PC9 | PC9GR |
| --- | --- | --- | --- | --- |
|  |  |  |  |  |
| AmeL | X, X | X, X | X, X | X, X |
| TH01 | 9.3,9.3 | 7,7 | 7,7 | 7,7 |
| D5S818 | 11,11 | 11,12 | 11,11 | 11,11 |
| D13S317 | 11,11 | 10,10 | 8,8 | 8,8 |
| D7S820 | 8,9 | 8,11 | 10,11 | 10,11 |
| D16S539 | 11,12 | 9,12 | 9,9 | 9,9 |
| CSFIPO | 11,11 | 12,12 | 11,11 | 11,11 |
| vWA | 18,18 | 18,18 | 17,17 | 17,17 |
| TPOX | 11,11 | 8,11 | 11,11 | 11,11 |
| Matched to | CRL-5883-NCI-H1650 | CRL-5908-NCI-H1975 | PC9 | PC9 |
