# Supplementary material for: Serine-arginine protein kinase 1 (SRPK1) promotes EGFR-TKI resistance by enhancing GSK3β Ser9 autophosphorylation independent of its kinase activity in non-small-cell lung cancer
Source: Oncogene. 2023 Mar 3;42(15):1233–46. doi: 10.1038/s41388-023-02645-2 (PMC10079535; doi:10.1038/s41388-023-02645-2)
Supplement: Supplementary file 11 — Table S3 [file 41388_2023_2645_MOESM11_ESM.docx]

**Table S3: The antibodies that used for WB and IHC**

| Antibodies | Source | Identifier | dilution |
| --- | --- | --- | --- |
| SRPK1 | BD Biosciences | cat. no. 611072 | 1:2000 for WB |
|  |  |  | 1:50 for IHC |
| β-catenin | BD Biosciences | cat. no. 610154 | 1:2000 for WB |
| EGFR | Cell Signaling Technology | cat. no. 4267 | 1:1000 for WB |
| p-EGFR-Y1068 | Cell Signaling Technology | cat. no. 3777 | 1:1000 for WB |
| Na+/K+ ATPase | Zen Bioscience | cat. no. 380790 | 1:5000 for WB |
| GSK3β | Abcam | cat. no. ab32391 | 1:5000 for WB |
| p-GSK3β-Ser9 | Abcam | cat. no. ab75814 | 1:5000 for WB |
|  |  |  | 1:100 for IHC |
| p−β-catenin S33/37 | Abcam | cat. no. 11350 | 1:1000 for WB |
|  |  |  | 1:100 for IHC |
| Bcl-xL | Abcam | cat. no. ab32370 | 1:1000 for WB |
| Bcl-xS | Thermo Fisher Scientific | cat. no. 32203 | 1:2000 for WB |
| Caspase-3 | Abcam | cat. no. ab184787 | 1:2000 for WB |
| Cleaved-caspase-3 | Abcam | cat. no. ab2302 | 1:500 for WB |
|  |  |  | 1:20 for IHC |
| Cleaved-PARP1 | Abcam | cat. no. ab32064 | 1:1000 for WB |
| PARP1 | Abcam | cat. no. ab227244 | 1:1000 for WB |
| α-tubulin | Abcam | cat. no. ab 7291 | 1:5000 for WB |
| β-tubulin | Abcam | cat. no. ab210797 | 1:5000 for WB |
| Histone 2A (H_2_A) | Abcam | cat. no. ab18255 | 1:1000 for WB |
| Goat Anti-Rabbit IgG H&L (HRP) | Abcam | cat. no. ab6721 | 1:5000 for WB |
| Goat Anti-Mouse IgG H&L (HRP) | Abcam | cat. no. ab6789 | 1:5000 for WB |
| LEF1 | Cell Signaling Technology | cat. no. 2569 | 1:1000 for WB |
| HA | Sigma | cat. no. H3663 | 1:1000 for WB |
| Flag | Sigma | cat. no. F1804 | 1:1000 for WB |
| His | Sangon Biotech | cat. no. D199987 | 1:1000 for WB |
| GST | Sangon Biotech | cat. no. D199985 | 1:1000 for WB |
