# Supplementary material for: Serine-arginine protein kinase 1 (SRPK1) promotes EGFR-TKI resistance by enhancing GSK3β Ser9 autophosphorylation independent of its kinase activity in non-small-cell lung cancer
Source: Oncogene. 2023 Mar 3;42(15):1233–46. doi: 10.1038/s41388-023-02645-2 (PMC10079535; doi:10.1038/s41388-023-02645-2)
Supplement: Supplementary file 12 — Table S4 [file 41388_2023_2645_MOESM12_ESM.docx]

**Table S4: Primers used for real-time PCR**

| Gene | Primers (5′-3′) |
| --- | --- |
| *SRPK1* | Forward: ATGGAGCGGAAAGTGCTTG  Reverse: GAGCCTCGGTGCTGAGTTT |
| *BCL2L1* | Forward: GAGCTGGTGGTTGACTTTCTC  Reverse: TCCATCTCCGATTCAGTCCCT |
| *CCND1* | Forward: GCTGCGAAGTGGAAACCATC  Reverse: CCTCCTTCTGCACACATTTGAA |
| *MYC* | Forward: CGTCCTCGGATTCTCTGCTC  Reverse: CTTCGCTTACCAGAGTCGCT |
| *MCL1* | Forward: GGAGATTCCTGACCAGAACATTG  Reverse: CGACTGGGCTTTATCAAGACAT |
| *BIRC5* (Survivin) | Forward: AGGACCACCGCATCTCTACAT  Reverse: AAGTCTGGCTCGTTCTCAGTG |
| *EGFR* | Forward: TTGCATTGATAGAAATGGGCTGC  Reverse: TCCAGACAAGCCACTCACC |
| *CTNNB1* | Forward: GTGCAATTCCTGAGCTGACA  Reverse: CTTAAAGATGGCCAGCAAGC |
| *LEF1* | Forward: CAGTCATCCCGAAGAGGAAG  Reverse: AGGGCTCCTGAGAGGTTTGT |
