# Supplementary material for: Serine-arginine protein kinase 1 (SRPK1) promotes EGFR-TKI resistance by enhancing GSK3β Ser9 autophosphorylation independent of its kinase activity in non-small-cell lung cancer
Source: Oncogene. 2023 Mar 3;42(15):1233–46. doi: 10.1038/s41388-023-02645-2 (PMC10079535; doi:10.1038/s41388-023-02645-2)
Supplement: Supplementary file 13 — Table S5 [file 41388_2023_2645_MOESM13_ESM.docx]

**Table S5: Primers were used for plasmid construction**

| Gene | Primers (5′-3′) |
| --- | --- |
| *SRPK1* | Forward: ATGGAGCGGAAAGTGCTTGC |
|  | Reverse: TTAGGAGTTAAGCCAAGGGTGC |
| *GSK3B* | Forward: ATGTCAGGGCGGCCCAGA |
|  | Reverse: TCAGGTGGAGTTGGAAGCTGA |
| *SRPK1* (K109A) | Forward: TGTGGCAATGGCAGTAGTTAAAAGTGCTGAACATTACACT |
|  | Reverse: TTAACTACTGCCATTGCCACAAATTTCTTCCCCTGAAT |
| *GSK3B* (S9A) | Forward: GGTCGCCATCGCGAAAGTATTGCAGGACAAGAGATTTAAG |
|  | Reverse: AATACTTTCGCGATGGCGACCAGTTCTCCTGAATCACA |
| *GSK3B* (K85A) | Forward: AGAACCACCGCCTTTGCGGAGAGCTGCAAGCC |
|  | Reverse: CAAAGGCGGTGGTTCTGGGCCGCCCTGACAT |
| *SRPK1* (ΔK1) | Forward: CAGTACATTCGGAGGCTGGC |
|  | Reverse: TTAGGAGTTAAGCCAAGGGTGC |
| *SRPK1* (ΔK2) | Forward: ATGGAGCGGAAAGTGCTTGC |
|  | Reverse: GAGCTTTTCTGATTTTTTGG |
| *SRPK1 Spacer* | Forward: CAGTACATTCGGAGGCTGGC |
|  | Reverse: GAGCTTTTCTGATTTTTTGG |
